# Supplementary material for: Comprehensive profiling of retroviral integration sites using target enrichment methods from historical koala samples without an assembled reference genome
Source: PeerJ. 2016 Mar 28;4:e1847. doi: 10.7717/peerj.1847 (PMC4824918; doi:10.7717/peerj.1847)
Supplement: Article S1 [file peerj-04-1847-s001.docx]

**Statistical modeling of shared KoRV integration sites among koalas**

**Background**

As described in the main text, shared integration sites found in two individuals were inherited from their common ancestor, which means KoRV integrated into this locus of their common ancestor genome. Therefore, investigation of shared integration sites will reveal the integration history of KoRV. Furthermore, the comprehensive integration record of multiple proviruses in many koala individuals can elucidate the KoRV endogenization process. Based the comparison of integration sites from this study, Tsangaras et al 2014 [1] and Ishida et al 2015 [2], the sharing of integration sites among these 23 koalas increases over the past 140 years can be monitored using a statistic modeling, Generalised Mixed effect Model (GLMM). However, the low amount of shared integration sites between the three studies can be due to the different level of intensiveness for KoRV flanks targeting, which can potentially miss many shared integration sites. Therefore, a statistical modeling of shared KoRV integration sites over time when the koalas were sampled was only performed to the ten museum koalas in this study.

**Result:**

The proportion of 5’ integration sites that were shared with other koalas was significantly higher in more recently collected koala specimens than in specimens collected further in the past. This was true both when the influence of the identity of the koala and of the insertion site were accounted for in a statistical model (time effect in the Generalised Mixed effect Model: LRT=5.06, df=1, pv=0.0024), and on raw mean occurrence frequencies pooled across insertion sites (Spearman correlation test, rho=0.75, pv=0.033; Fig. 1 A). For the 3’ data set, the increase with time in raw mean occurrence frequencies pooled across integration sites did not reach significance (Spearman correlation test, rho=0.57, pv=0.15) due to the low prevalence of integration sites (7.53 %) observed in the koala sampled in 1960 (Figure 1 B). However, similar to the 5’ data set, the prevalence of shared integration sites did increase with time when controlling for the effect of koalas and insertion site (time effect in the GLMM: LRT=5.53, df=1, pv=0.019).

**Discussion:**

KoRV integrations demonstrate significant increased sharing of integration sites among museum koalas in the more recently collected samples. While DNA degradation may alter the detection of both shared and non-shared integrations, modern and historical koalas demonstrated a strong bias against shared integration sites. Moreover, the ancient DNA samples from the current data set did not demonstrate a linear pattern of poorer sample performance based on age. Therefore, data suggests that the proportion of KoRVs shared across koalas has increased in over a period of 110 years. Besides, Simmons et al. 2012 also provide evidence to support for fixing of KoRV through time.

As some of the samples, particularly the oldest were from New South Wales and the younger samples from Queensland, the results could also be explained by geographical differences in specific KoRV integrations. The more commonly shared integrations may represent older KoRV integrations that endogenized earlier and that have had more time for drift to increase their frequency in the population and their geographic extent within the koala population.

**Conclusion**

Although the statistic modelling showed a trend of significantly increased sharing of integration sites through time among the ten koalas, we should be cautious to address such a conclusion due to small number of koalas studied and the unclear genealogy between the ten koalas.

**Methods: Statistical analysis of shared integration sites**

Statistical tests were performed to check if the occurrences of KoRV at sampled integration sites increased as the samples became younger among the 10 museum koala samples. Two logistic regression models were employed: one for 5’ integration sites and one for 3’ integration sites. Both models had the same structure. The occurrence was considered (binary: 1=presence, 0=absence) as the response variable and time as a continuous fixed effect. Because results were qualitatively similar irrespective of expressing "time" as rank or directly as years, for the sake of simplicity, only the latter was reported. The identity of koalas and of insertion sites were considered as two Gaussian random effects, making this logistic regression a Generalised Mixed effect Model (GLMM). The GLMM was fitted using the function HLfit from the R package spaMM 1.4.1 (56), considering a Binomial error structure. The effect of time was tested by performing an asymptotic Likelihood Ratio Test (LRT) using the function anova.HLfit from the same packages.

**References:**

1. Tsangaras K, Siracusa MC, Nikolaidis N, Ishida Y, Cui P, Vielgrader H, Helgen KM, Roca AL, Greenwood AD: **Hybridization Capture Reveals Evolution and Conservation across the Entire Koala Retrovirus Genome**. *PLoS ONE* 2014, **9**:e95633.

2. Ishida Y, Zhao K, Greenwood AD, Roca AL: **Proliferation of Endogenous Retroviruses in the Early Stages of a Host Germ Line Invasion**. *Mol Biol Evol* 2014:msu275.

**Figure S2. The proportion of KoRV integration sites that are shared among koalas may be increasing over time.** The horizontal axis shows the year of collection of museum koala samples screened for KoRV. The vertical axis shows the proportion of KoRV integration sites within a koala sample that were also detected in other koalas. Dots connected by dashed lines represent the mean prevalence for each year of sampling. The full line represents the prediction from the statistical analysis (Generalised Mixed effect Model): it shows that every 20 years, the odd for shared KoRV integration sites increased by 1.26 times for the 5’ data set (LRT=5.06, df=1, pv=0.0024) and by 1.87 times for the 3’ data set (LRT=5.53, df=1, pv=0.019), among the ten koala specimens examined.
