# Supplementary figures and images for: Comprehensive profiling of retroviral integration sites using target enrichment methods from historical koala samples without an assembled reference genome
Source: PeerJ. 2016 Mar 28;4:e1847. doi: 10.7717/peerj.1847 (PMC4824918; doi:10.7717/peerj.1847)

# Sequence length distribution of aDNA samples

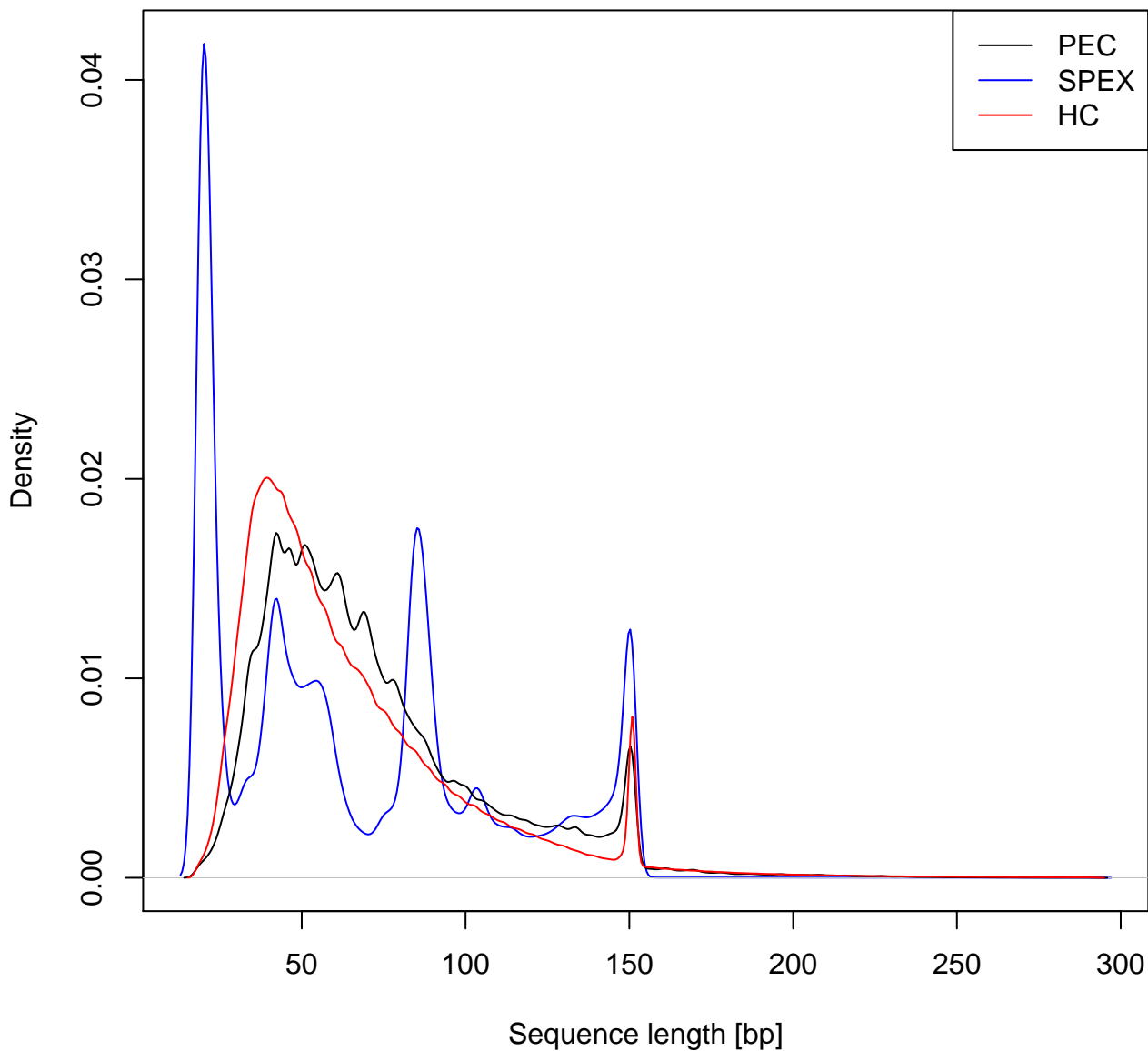

Supplement: Figure S1 — R plot of sequence length distributions from PEC (red), SPEX (blue) and hybridization capture (HC) (green) reads. [file peerj-04-1847-s002.pdf]

A.

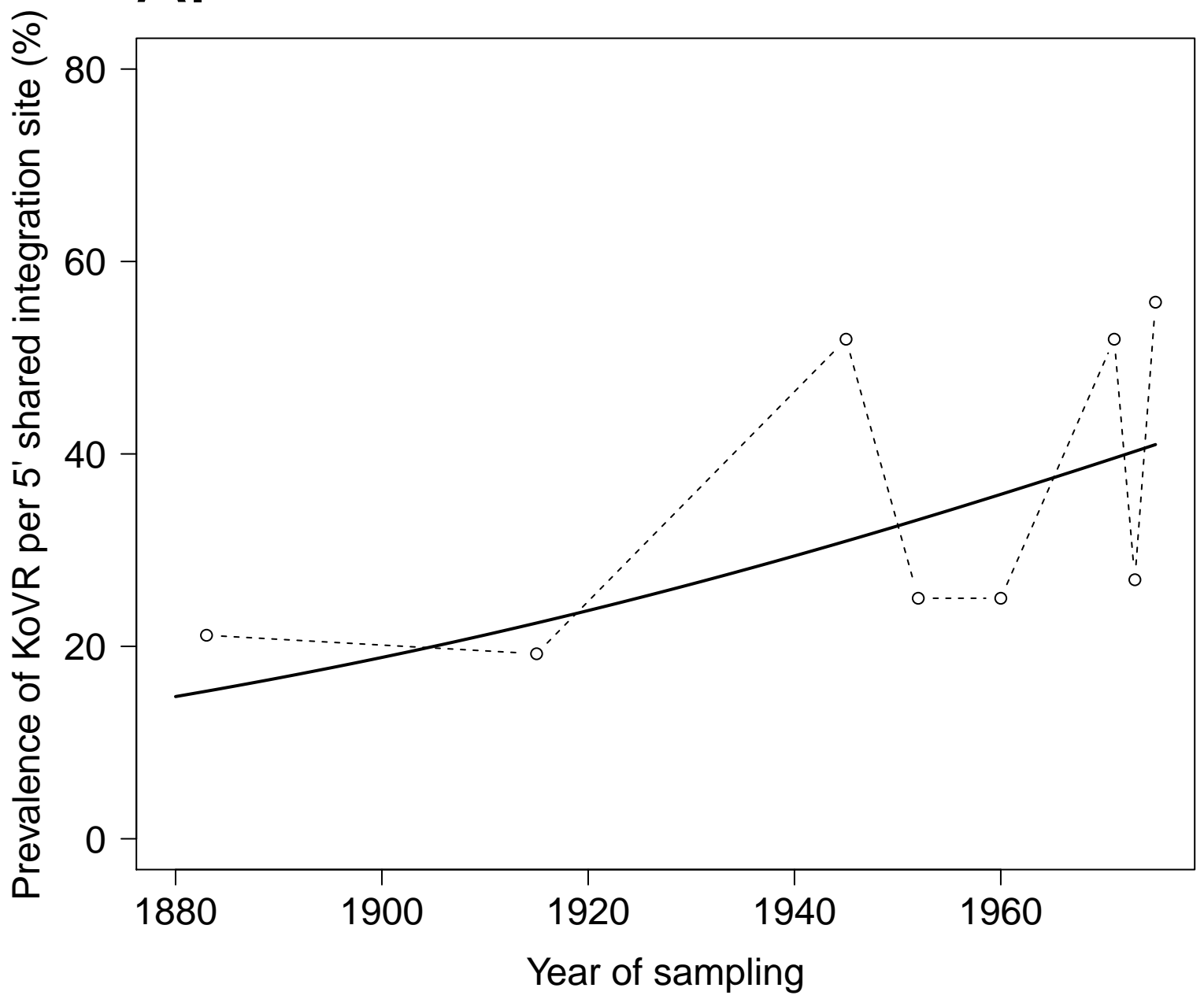

B.

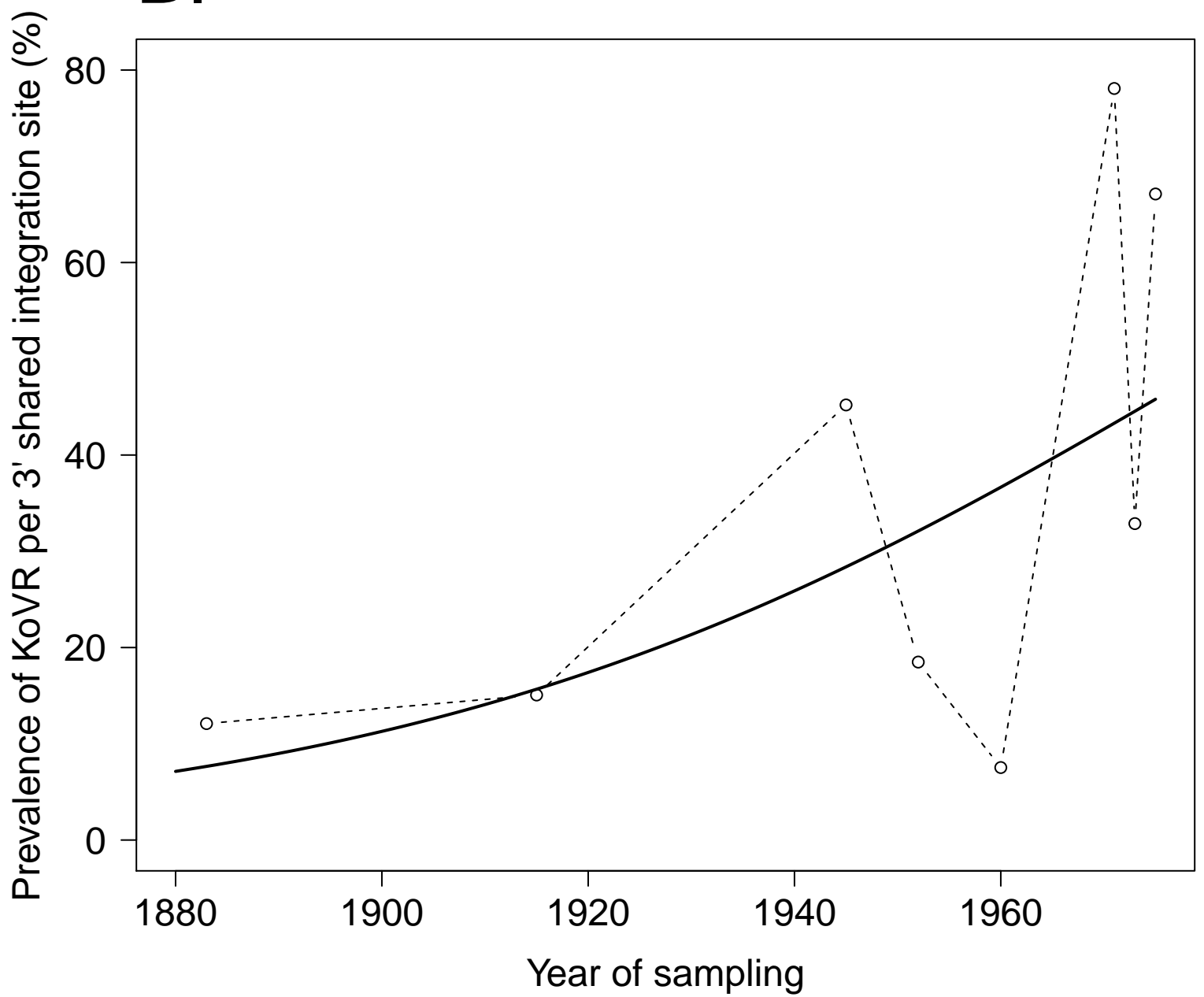

Supplement: Figure S2 — Plotted results of a Generalised Mixed effect Model analysis against the shared integration sites by year. [file peerj-04-1847-s003.pdf]
