# Supplementary material for: Comprehensive profiling of retroviral integration sites using target enrichment methods from historical koala samples without an assembled reference genome
Source: PeerJ. 2016 Mar 28;4:e1847. doi: 10.7717/peerj.1847 (PMC4824918; doi:10.7717/peerj.1847)
Supplement: Data S1 — The sequences shown are the concatenation of matched 5′ and 3′ integration sites from a single koala individual. [file peerj-04-1847-s004.pdf]

Matched pairs of 5’ and 3’ integration sites that come from the same koala.

>P_3rU_6s8_cluster_446_cluster_937__C_5fU_S8.120509_S8.334494

GAATTAGAAATCGAGGAGATGCCCATCAATTGGGGAATGGCTGAACAAGTCATG
GTATAAGGAATTATGATGTGATGTTGTCTTCATGATCCCATTTAGAGTTTTCTTG
GCAAAGA

>P_3rU_cluster_564_S_3fS_cluster_29_cluster_977_cluster_4_cluster_n1_cluster_2
8__C_5fU_2S3_S_5rS_cluster_7_C_5rS_S3.746665_S4.29352_S_S9.36057_S1.30
664_S5.12084_S10.22389

GAACCCACAAAAAGACAGAATGAAACAAATATCCAGCCCAATACAGCCTGGATG
ATTGATCAGAAGGGTGTATCGCGC CAGGCTGGGAGCACAG

>S_3rU_S17.112853_P_3rU_8S2__P_5fS_cluster_35

GTAAATTGGTCTGAGGCTGGATTTGAACTCAGATCCTCCTGACT

>P_3rU_S8.18869_S8.19758_S8.24709__P_5fU_cluster_54_C_5fU_2S8

AGTGGCTTGCCCCGGGGCACACAGCTAGTATGTATCGGAGGCTGGATTTGAAC
TCAGGT

>P_3rS_cluster_175.maffT1s4-6s8__P_5fU_cluster_54_C_5fU_2S8

AGGAAACTGAGGCAAAGTTAAGTGATTTGCCGGGTCACACAGCTAGTATGTATC
GGAGGCTGGATTTGAACTCAGGT

>P_3rU_S2.133819_S2.154135__P_5fU_S2.2639_C_5fU_S2.23072_S2.547057

GACAGCATTTTCCATCCTGCGGCCTCTGGAGATGTCTTAGATCCTTGTATTGCT
GA

>P_3rS_S10.119113_7S2_P_3fU_S2.18246_S_3fS_cluster_68_S_3rU_S15.6385__
P_5fU_S2.2639_C_5fU_S2.23072_S2.547057

TATGCTTCAATCTACATTCAAACTCCGTAGTTCTTTCTTTGGATGTGGATAGCATT
TTTCATCATGAGGCCTTTGGAGATGTCTTAGATCCT TGTATTGCTGA

>P_3rS_cluster_31_cluster_252_P_3fS_S2.18623_S2.14939_S_3rU_2S17__P_5fU
_S2.2639_ C_5fU_S2.23072_S2.547057

CTTTTCCGATTTGACAGCATTTTCCATCCTGAGGCCTCTGGAGATGTCTTAGATC
CTTGTATTGCTGA

>P_3rU_2S8_P_3fU_7S8__C_5fU_3S8

CCGATCTTTGCCTGAGGTTGCACACTTGGTCATATTTGAGCTCAGGCAACTGGG
GTTAAGTGACTTGCCCAGAGTCACAAGTCTGAGGT CGGATTTGAA

>P_3rU_S8.12674_S8.39898__C_5fU_S8.950860

GACACCTCGGTGTGTCTCAGTTTCCTCATCTATAATATGAGCTGGAAAAGGAAAT
GGCACACTACTCTAGTATCTT TGCCAAGAAAACCC

>P_3rU_7S3__C_5fU_S3.480683

TATTGTGTCATGTAACCATATAGTATCTCATTGTAAACAAATCATTACATGCACAC
ATTCCCACCAATGCATGCTGGACTTCCTGACAAAGTACAACATGCTCACCTGCC
AACACTTGCTTGTTAAGACCTGTCAGTGGACTTGACCACTGATGGGTTATAGCTT
GCATTT

>P_3rU_cluster_231__C_5fU_2S8

ATTCAGCCTCAGACACTTTCCAGCTGTGTGACCCTGGGCAAGTCAGTTAACCCC
GTCTGCCTCAGTTTCTCCATCCATAAAATGAGCTGGAGAAAGAAATGGCAAACC
ACTCCAGGATCTTTGCCAAGAAATCCCCAAATGGGG

>P_3rU_S8.36760__C_5fU_S8.958388

CATATCAAATGACTTGTCCACAGTCACACAGC

>P_3rU_S8.36760__C_5rU_4S8

CATATCAAATGACTTGTCCACAGTCACACAGCTAGTTTCAGAGGTGAGATTTGAA
>P_3rU_cluster_446_cluster_937__P_5rU_3S8_C_5rU_S8.15867
GAATTAGAAATCGAGGAGATGCCCATCAATTGGGGAATGGCTGAACAAGTCATG
GTATAAGGAATTATGATGTGA

>P_3rU_cluster_446_cluster_937__C_5rU_S8.170262

GAATTAGAAATCGAGGAGATGCCCATCAATTGGGGAATGGCTGAACAAGTCATG
GTATATGAATGTAATGGAATACTATC GTGCTATAAGAAAGA

>P_3rS_8S3_S8.47689__S_5rU_cluster_541_C_5rU_S8.50529

ATACACATAAATTAGAGATAAGAGGCAGAGTTGCACAGTCATCAGCCTCACTTTC
TC

>P_3rU_4S8_P_3fU_S8.70215__C_5rU_S8.1022487

TTAAAGACAGCAATCTTCTGTCTATTCTTCAAGATTGGTTTCAGGAAACTTTAACT
GGGGGCTGGAAAAGCATCCCAT CATTTCTGACTTCCATCCTCCTTCTACTG

>P_3rU_6s3_P_3fS_3S2_2S9_S8.5461_S5.1115_2S4_3S3_S_3fS_cluster_29_cluster_977_cluster_4_cluster_n1_cluster_28__S_5rS_clsuter_cp1_C_5fU_2S7_P_5fU_cluster_38_C_5rS_S7.10526_2S10_2S3_S4.17612_S5.4886_S9.1880_S1.11056_2S8_S2.123759

GAACCCACAAAAAGACAGAATGAAACAAATATCCAGCCCAATACAGCCTGGATGATTGATCAGAAGGGTGTATCGCGCCTTGCTAGGAGCGCAGTGCAGCGCGGTGTGGGCGCACAGGCTGCAGCAAACCTGGAGCAGGCCTCAGACTGAATCATGGGCAGCTG

>P_3rU_6s3_P_3fS_3S2_2S9_S8.5461_S5.1115_2S4_3S3_S_3fS_cluster_29_cluster_977_clu ster_4_cluster_n1_cluster_28__S_5rU_S20.31638_C_5rU_S3.50689

GAACCCACAAAAAGACAGAATGAAACAAATATCCAGCCCAATACAGCCTGGATGCTTGATCGGA

>P_3rS_cluster_31_cluster_252_P_3fU_3S2_S_3rS_2S17_S15.6385_S_3fS_cluster_68__S_5rU_S18.27629_C_5fU_S2.1235481_C_5rU_S2.466916

GACAGCATTTTCCATCCTGAGGCCTCTGGAGATGTCTTAGATCCTTGTATTGCTGAGAAGGGTTAAGTCTATTAATATTAGTACCTAACTGATTATGTTATTCTCTTCTTGAGCCAAATCTGATGAGAGTAAGGTTCAAACAATGCTAATATCCGTC

>P_3rS_S5.47458_6S8__S_5rS_cluster_43_C_5fU_S8.854973

ATAAATGAGGAACCTGATATCCAAAGAACTGAAATGACTTACCAAGGTCACACAGCTGATGAGTAGCAGAAGCAAGAAGAGAAACAAAATCTTCTGATTCCCAGGTTCCTGCCAC

>P_3rU_cluster_231__C_5rU_S8.503819
ATTCAGCCTCAGACACTTTCCAGCTGTGTGACCCTGGGCAAGTCAGTTAACCCCGTCTGCCTCAGTTTCC

>P_3rU_S8.12674_S8.39898__C_5rU_S8.911698

GACACCTCGGTGTGTCTCAGTTTCCTCATCTATAAAATGAGCTGGAGAAGGAAATGACAAACCACTCTAGTATCTTTGCCAAGAAAACCCCAAATGAGATCA

>P_5fU_cluster_54.maffT3s8_C_5fU_S8.30495_S8.561570__P_3fU_S8.88040_S8.3563

ACCTGAGTTCAAATCCAGCCTCCGATACATACTAGCTGTGCGACCCGGGGCAAGCCACT

>P_5fU_cluster_54.maffT3s8_C_5fU_S8.30495_S8.561570__P_3fU_6S8_3fU_S8.1
05580

ACCTGAGTTCAAATCCAGCCTCCGATACATACTAGCTGTGTGACCCGGGGCAAG
CCACT

>P_5fU_S2.26104_C_5fU_S2.82531__P_3fU_S2.2953

CTGGGAGTTAGGGAGGACCTGAGTTCAAATCCAGCCTCAGACACATAACACTTA
GCATATGTGATG

>C_5fU_S2.410158__S_3fU_S18.5221_P_3fU_S2.310

CATTTTTATTTATTCATACATACTTCCAATCATCAATATGAGAACCATTTTATGTGC
AATACATTGTGCTTTCCAGAACAGTGGAGCCAATTCCCAGCTCCACCAACAATG
CATCAGTG

>C_5fU_S8.589094__C_3fU_S8.917292_P_3fU_S8.49463

CCCAAGAAGTGGTATTGCTGGATCAAAGGGTATGCAGTTTTATAGCCCTTTGGG
CATAGTTCCAAAT

>C_5fU_S3.480683__P_3fU_S3.28583_S8.82168_S3.90397_S3.64168_S_3fU_S20
.10443

AAATGCAAGCTATAACCCATCAGTGGTCAAGTCCACTGACAGGTCTTAACAAGC
AAGTGTTGGCAGGTGAGCATGTTGTACTTTGTCAGGAAGTCCAGCATGCATTGG
TGGGAATGTGTGCATGTAATGATTTGTTTACAATGAGATACTATATGGTTACATG
ACACAATATTGTGTCATCAAAATTTTGATGCAGGGGAAAAACTCACAAATTTACA
ATAAATT

>C_5fU_S8.92543_S8.302794__P_3fU_S8.42372

CCCCATTTGGGGATTTCTTGGCAAAGATCCTGGAGTGGTTTGCCATTTCTTTCTC
CAGCTCATTTTATGGATGGAGAAACTGAGGCAGACGGGGTTAACTGACTTGCCC
AGGGTCATACAACTAGGAAGTGTCTGAGGCCAGATTTGAATCCAAGAAGATAAG
TCCTCCTGACTCCGGGTTTGGCAGTCTGTCCACTATGACA

>C_5fU_S3.102282_S3.954929__S_3fU_cluster_195_P_3fU_S3.39389

ATATTATATTCCATGCCCAGCGGTCCTTTAATGTAGAAGCTGCTAAAACTTGTGT
TATCCTGATTGTGTTTCCACTATACTTGAATTGTTTCTTTCTTGCAGCTTGTAATA
TAT

>C_5fU_S8.242952_S8.249062__S_3fU_S17.14523_C_3fU_S8.70423_C_3rU_clust
er_339

TGCTTGGAACCATCGGTTATAGCAAATGGAGAAGTTTTGAACTCTGTGGATAAGT
TCACTTACCTCGGTAGTGTACTA

>P_5rU_cluster_19_C_5rU_S8.10722__S_3fU_S17.14523_C_3fU_S8.70423_C_3r
U_cluster_3 39

TTTCTCCACCAGCTGGCACCACATCATCTATGCTTGGAACCATCGGTTATAGCAA
ATGGAGAAGTTTTGAACTCT GTGGATAAGTTCACTTACCTCGGTAGTGTACTA

>C_5rU_2S3__S_3fU_4S20_P_3fU_S3.74019

GGCCGGTGCTCTATTCACTGTGCCACCTAGATGCCCCTGAAGAATATATTTTAG
GCATATAAATGTGTAT

>C_5rU_S2.906334__P_3fS_5S3_S2.110884_S8.68922

GGTCACCCAGCTAGTAAATATCTGAGGCCAGATTTGAGTCTTTCTGACTTCAGG
CCCTGCACTTTATTCACTGTGCCACCTAGATGAGATCG

>S_5rU_cluster_645.maffT2s20_C_5rU_S3.16848__S_3fU_S20.29912

GACCAGACTGATTAGAAGCATAACTACAAAATTCTGATTATTGCAATAACCCTTA
AGTATAATATTCCAATTAAGACA TCCAAGAGTCACATTTAAATATTGCACTCTTC

>C_5rU_S8.108028__P_3fU_S8.380

GAATGGGGCACAGTAGTAATAACATTAAAAAGACACACAACTTTGAGAGAATTAA
GGACTTTGATCAACCTAATGACTAACCACAGTTCCAG

>C_5rU_S8.490159__P_3fU_S8.380

GAAGGAGCAGAAATAACATTAAAAAGACACACAACTTTGAGAGAATTAAGGACTT
TGATCAACCTAATGACTAACCACAGTTCCAG

>C_5rU_S8.503819__P_3fU_S8.42372

GGAAACTGAGGCAGACGGGGTTAACTGACTTGCCCAGGGTCATACAACTAGGA
AGTGTCTGAGGCCAGATTTGAATCCAAGAAGATAAGTCCTCCTGACTCCGGGTT
TGGCAGTCTGTCCACTATGACA
