# Supplementary material for: Comprehensive profiling of retroviral integration sites using target enrichment methods from historical koala samples without an assembled reference genome
Source: PeerJ. 2016 Mar 28;4:e1847. doi: 10.7717/peerj.1847 (PMC4824918; doi:10.7717/peerj.1847)
Supplement: Data S2 — The sequences shown are the concatenation of matched 5′ and 3′ integration sites from different koala individuals. [file peerj-04-1847-s005.pdf]

Matched pairs of 5’ and 3’ integration sites that come from different koalas.

>P_3rS_S2.74718_S3.57590_11S8__C_5fU_3S8

TATTTGAGCTCAGGCAACTGGGGTTAAGTGACTTGCCCAGAGTCACAAGTCTGA GGTCGGATTTGAA

>P_3rU_4S8_P_3fU_S8.70215__C_5fU_S4.684182_5fU_S4.1661030

TTAAAGACAGCAATCTTCTGTCTATTCTTCAAGATTGGTTTGCCATTTCCTTCTCC AGCTCATTTTATGGAT

>P_3rU_4S8_P_3fU_S8.70215__C_5fU_S4.1683056

TTAAAGACAGCAATCTTCTGTCTATTCTTCAAGATTGGTTTCCTCATCTGTAAAAT GGGGATAATAACA

>P_3rU_S3.26225__C_5fU_S8.589094

AATTGAGGAACTATGCCCAAAGGGCTATAAAACTGCATACCCTTTGATCCAGCA ATACCACTTCTTGG G

>P_3rU_S8.97760__C_5fU_S3.480683

ACTGAGCCATATAACCATATAGTATCTCATTGTAAACAAATCATTACATGCACAC ATTCCCACCAATGCATGCTGGACTTCCTGACAAAGTACAACATGCTCACCTGCC AACACTTGCTTGTTAAGACCTGTCAGTGGACTTGACCACTGATGGGTTATAGCTT GCATTT

>P_3rS_S5.105847_5S8__C_5fU_S4.1481035

TGAATGAACATTTTCTCTACTCCGCCATCTTGGCTCCACCCCCC

>C_3rU_S8.1018030_P_3rS_S4.143028_6S3_S2.99154_17S8_P_3fU_2S8_S_3fU _S20.58049__P_5rU_S9.30145

GGCTGATTCGGACTCAGGTGAGTCTTCCAACTCCAGGGCTGGCACTCTATCCAT TCCCCCACCTACCTGCCCTCCCCACATTCCTTC AAACCCTCTGTC

>P_3rS_S3.41285_S4.156475_S5.66477_S7.113364_S8.73375_S9.5274_36S2__S _5rU_S18.20949_C_5rU_S2.111871

AGGAGGTTGAACCAGATGACCTCTGGGGTCTCTTTTAGCC

>S_3rU_S17.112853_P_3rU_8S2__S_5rU_S23.5584_2S20_C_5rS_3S3_S7.4344

GTAAATTGGTCTGAGGCTGGATTTGAACTCAGGTC

>P_3rU_4S8_P_3fU_S8.70215__C_5rU_S4.1173169

TTAAAGACAGCAATCTTCTGTCTATTCTTCAAGATTGGTTTGTCATTTCCTTCTCC
AGCTCATG

>P_3rS_S2.74718_S3.57590_11S8__C_5rU_S9.964451

TATTTGAGCTCAGGCAACTGGGGTTAAGTGACTTGCCAGATCGGAAGAGCGT

>C_5fU_S2.547057__P_3fU_S4.4833

TCAGCAATACAAGGATCTAAGACATCTCCAA

>C_5fU_S8.1066112_S8.1022169_S8.1055816__S_3fU_S20.2502

TTCAAATCCGACCTCAGACTTGTGACTCTGGGCAAGTCACTTAACCCCAGTTGC
CTCAGATCCAATTCACAT

>C_5fU_S4.684182_5fU_S4.1661030__S_3fU_S17.64559_P_3fU_S8.56518

ATCCATAAAATGAGCTGGAGAAGGAAATGGCAAACTAGTTTCCCAGTCTATTTCA
TCCGGAGTTGT

>C_5fU_S4.1683056__S_3fU_S17.64559_P_3fU_S8.56518

TGTTATTATCCCCATTTTACAGATGAGGAAACTAGTTTCCCAGTCTATTTCATCC
GGAGTTGT

>C_5fU_S2.729164__S_3fU_4S20_P_3fU_S3.74019

CTTTATTCACTGTGCCACCTAGATGCCCCTGAAGAATATATTTTAGGCATATAAA
TGTGTAT

>C_5fU_S4.1481035__P_3fU_S8.33956

GGGGGGTGGAGCCAAGATGGCGGAGTAGAAGATGAGGAAAAGGCTCACAGAA
GG

>S_5rU_cluster_620_C_5rU_2S8_P_5rU_S8.78052__P_3fU_S3.22152

AGAGGTCCAGGCAAAGAGCTATGATCCCCGGTTTCTGCTTTCCTTCTAGTTAAA
TCGGA

>C_5rU_S2.906334__S_3fU_4S20_P_3fU_S3.74019

GGTCACCCAGCTAGTAAATATCTGAGGCCAGATTTGAGTCTTTCTGACTTCAGG
CCCTGCACTTTATTCACTGTGCCACCTAGATGCCCCTGAAGAATATATTTTAGGC
ATATAAATGTGTAT

>C_5rU_2S3__P_3fS_5S3_S2.110884_S8.68922

GGCCGGTGCTCTATTCACTGTGCCACCTAGATGAGATCG

>C_5rU_S4.1173169__S_3fU_S17.64559_P_3fU_S8.56518

CATGAGCTGGAGAAGGAAATGACAAACTAGTTTCCCAGTCTATTTCATCCGGAG
TTGT

>S_5rU_S23.5584_2S20_C_5rS_3S3_S7.4344__P_3fS_8s2_1s5

GACCTGAGTTCAAATCCAGCCTCAGACAAATTCCAAGAAAAAGTTAGCTCTTTCC
CCTTCCTCCCCCTC CTGTGCCAT

>P_5rU_S4.11115__S_3fU_S20.10939_P_3fU_S3.74062

TTCAAGCAGAAGACGGCATACGAGATAGAGGCGGTGACTGGAGTTCAGACGTG
TGCTTTGCCGATCTGAAGACACAGTAGTCAATGACTATAGTAGTCTTC
